# Enteral versus parental nutrition after pancreaticoduodenectomy under enhanced recovery after surgery protocol: study protocol for a multicenter, open-label randomized controlled trial (ENE-PAN trial)

**DOI:** 10.1186/s13063-022-06856-y

**Published:** 2022-10-29

**Authors:** Yoshinori Takeda, Yoshihiro Mise, Yoji Kishi, Hiroyuki Sugo, Yusuke Kyoden, Kiyoshi Hasegawa, Yu Takahashi, Akio Saiura

**Affiliations:** 1 Department of Hepatobiliary-Pancreatic Surgery, Juntendo University School of Medicine, 2-1-1 Hongo, Bunkyo-Ku, Tokyo, 113-8421 Japan; 2 Department of Surgery, National Defense Medical College, Tokorozawa, Saitama Japan; 3 Department of General Surgery, Juntendo University Nerima Hospital, Nerima, Tokyo, Japan; 4 Department of Gastrointestinal Surgery, Ibaraki Prefectural Central Hospital, Koibuchi, Kasama, Ibaraki Japan; 5 Hepato-Biliary-Pancreatic Surgery Divison, Department of Surgery, Graduate School of Medicine, University of Tokyo, Bunkyo-Ku, Tokyo, Japan; 6 Division of Hepatobiliary and Pancreatic Surgery, Cancer Institute Hospital, Japanese Foundation for Cancer Research, Ariake, Tokyo, Japan

**Keywords:** Pancreaticoduodenectomy, Enteral nutrition, ERAS

## Abstract

**Background:**

Infectious complications are the main causes of morbidity after pancreaticoduodenectomy (PD). Early enteral nutrition (EN) is a reasonable form of nutritional support that aims to mitigate the occurrence and severity of infectious complications by maintaining gut immunity. However, it remains unclear whether EN is beneficial for patients who underwent PD and are under enhanced recovery after surgery (ERAS) protocol.

**Methods:**

A multicenter (six hospitals), open-label, randomized controlled trial will be started in July 2022. A total of 320 patients undergoing open PD will be randomly assigned to an EN group or a peripheral parental nutrition (PPN) group in a 1:1 ratio. The stratification factors will be the hospital, age (≥ 70 or not), and preoperative diagnosis (pancreatic cancer or not). In the EN group, enteral nutrition will start on postoperative day (POD) 1 at 200–300 ml/day via the percutaneous tube placed operatively. The volume of the diet will be increased to 400–600 ml/day on POD 2 and depend on the surgeon’s decision from POD 3. In the PPN group, PPN will be delivered after surgery. In both groups, oral feeding will start on POD 3. Each treatment will be finished when patients’ oral food intake reaches 60% of the nutritional requirement (25–30 kcal/day). The primary endpoint will be the occurrence of postoperative infectious complications within 90 days of surgery. The secondary endpoints will be all complications, including major ones such as Clavien–Dindo grade 3 or more and clinically relevant postoperative pancreatic fistula. Data will be analyzed per the intention to treat.

**Discussion:**

This will be the first, large, and well-designed RCT that aims to determine whether EN is beneficial for patients who underwent PD under the ERAS protocol. According to the results of this study, either EN or PPN would be adopted as the standard nutritional support for patients undergoing PD.

**Trial registration:**

jRCT1030210691. Registered on March 23, 2022.

## Administrative information

Note: the numbers in curly brackets in this protocol refer to SPIRIT checklist item numbers. The order of the items has been modified to group similar items (see http://www.equator-network.org/reporting-guidelines/spirit-2013-statement-defining-standard-protocol-items-for-clinical-trials/).

**Table:** 

Title {1}	Enteral versus parental nutrition after pancreaticoduodenectomy under enhanced recovery after surgery protocol; study protocol for a multicenter, open-label randomized controlled trial
Trial registration {2a and 2b}	jRCT1030210691
Protocol version {3}	Feb. 25, 2022 Version 1.0
Funding {4}	The authors received no specific grant from any funding agency in the public, commercial, or not-for-profit sector for this study
Author details {5a}	Yoshinori Takeda^1^, Yoshihiro Mise^1^, Yoji Kishi^2^, Hiroyuki Sugo^3^, Yusuke Kyoden^4^, Kiyoshi Hasegawa^5^, Yu Takahashi^6^, Akio Saiura^1^ ^1^Department of Hepatobiliary-Pancreatic Surgery, Juntendo University School of Medicine, Hongo, Tokyo, Japan ^2^Department of Surgery, National Defense Medical College, Tokorozawa, Saitama, Japan ^3^Department of General Surgery, Juntendo University Nerima Hospital, Nerima, Tokyo, Japan ^4^Department of Gastrointestinal Surgery, Ibaraki Prefectural Central Hospital, Koibuchi, Kasama, Ibaraki, Japan ^5^Hepato-Biliary-Pancreatic Surgery Divison, Department of Surgery, Graduate School of Medicine, University of Tokyo, Bunkyo-Ku, Tokyo, Japan ^6^Division of Hepatobiliary and Pancreatic Surgery, Cancer Institute Hospital, Japanese Foundation for Cancer Research, Ariake, Tokyo, Japan
Name and contact information for the trial sponsor {5b}	Not applicable
Role of sponsor {5c}	Not applicable

## Introduction

### Background and rationale {6a}

Pancreaticoduodenectomy (PD) is a high-risk surgical procedure for patients with pancreatic head cancer and distal bile duct cancer. Despite advancements in surgical techniques and perioperative management, the morbidity rate remains high at 65–71% [1, 2]. Infectious complications, including postoperative pancreatic fistulas (POPFs) and organ surgical site infection, are the main causes of the morbidity and are sometimes life-threatening [3, 4]. To mitigate the occurrence and severity of infectious complications, several approaches, such as nutritional support, have been evaluated in many studies [1, 5–7].

Enteral nutrition (EN), which is easy to perform, is the most recommended postoperative artificial nutritional support for patients who undergo PD [8]. For patients with catabolic stress such as those who just underwent major surgery, early feeding via the enteral route could be a proactive therapeutic strategy that reduces the rate of infectious complications by helping to favorably modulate the immune response [9]. The underlying beneficial mechanism of EN is the presence of a physiological and immunological barrier, which could be impaired rapidly by starvation (“nil by mouth”) even when patients receive adequate parental nutrition [10].

Therefore, early EN was identified as reasonable management to mitigate the occurrence of infectious complications after PD; however, its benefits have been controversial so far. Previously, Grizas reported that early EN decreased the rate of infectious complications after PD more than peripheral parenteral nutrition (PPN) did [11]. On the other hand, Perinel showed that EN did not decrease the infectious complication rate more than total parenteral nutrition (TPN) did and that EN was associated with increased overall morbidity [1]. However, these randomized controlled trials (RCTs) had some problems in their sample sizes or postoperative management execution, such as the delayed restart of oral feeding. Recently, enhanced recovery after surgery (ERAS), including the early restart of feeding that aims to shorten the patient’s hospital stay without increasing the morbidity or readmission rates, has been accepted as standard perioperative management for patients undergoing PD [12]. This management could minimize the duration of starvation and prevent the impairment of gut immunity. Therefore, we designed a multicenter RCT to determine whether EN decreases the infectious complication rate after PD more than PPN does under the ERAS protocol.

### Objectives {7}

This study aims to investigate whether EN reduces the rate of postoperative infectious complications in patients undergoing PD and the ERAS protocol.

### Trial design {8}

The ENE-PAN trial is a randomized controlled, parallel-group, open-label, multicenter, superiority trial investigating the effectiveness of EN versus PPN after PD under the ERAS protocol. Eligible patients will be randomized equally to either the EN or the PPN group.

## Methods: participants, interventions, and outcomes

### Study setting {9}

**Fig. 1:**
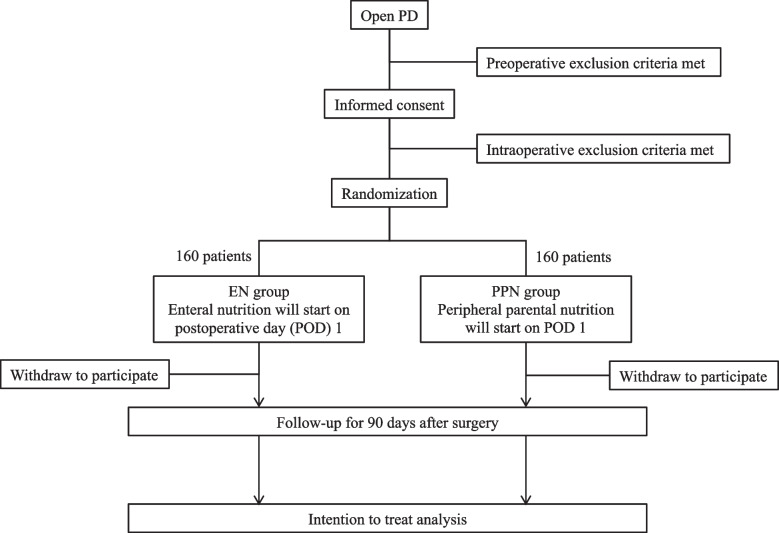
Consolidated Standards of Reporting Trials (CONSORT) flowchart. PD, pancreaticoduodenectomy; EN, enteral nutrition; PPN, peripheral parental nutrition

The study is an investigator-initiated, multi-institutional, two-arm, open-label randomized trial in six Japanese academic hospitals. The flowchart of the trial is shown in Fig. 1.

### Eligibility criteria {10}

The inclusion criteria are below:

-
  - Age ≥ 20 years
  - Indication for elective PD
  - American Society of Anesthesiologists performance score of 1–3
  - Written informed consent given by the patient

The exclusion criteria are as follows:

-
  - Robot-assisted or laparoscopic PD
  - Combined liver resection
  - Antibiotic therapy within a week before surgery
  - Contraindications for EN (allergy, disorder of amino acid metabolism, etc.)
  - Severe cardiac, liver, or renal comorbidities
  - Patient judged to be ineligible for enrolment by the investigator

### Who will take informed consent? {26a}

Potential participants will be identified from patients who visit hospitals. After assessment by surgeons regarding inclusion and exclusion criteria, the trial information will be given to potential participants in person by surgeons. Written and verbal informed consent will be obtained. The right of a participant to refuse to participate in the study without giving reasons for the decision will be respected.

### Additional consent provisions for collection and use of participant data and biological specimens {26b}

Patients included in the study will be offered the opportunity to participate in an ancillary study investigating the survival of patients. Consent for participating in the ancillary study will be obtained when the study starts.

## Interventions

### Explanation for the choice of comparators {6b}

There is poor evidence as to which form of nutritional support is the best at present. The international study group of pancreatic surgery recommends the early resumption of oral intake in the ERAS protocol [8]. According to a review article, early mandatory postoperative TPN is not associated with improved outcomes [13].

### Intervention description {11a}

The patients included in the study will undergo open PD on day 0 and be randomized to a PPN (control) group or an EN (experimental) group.

In the PPN group, PPN will be delivered after surgery. In the EN group, EN will start on postoperative day (POD) 1 at 200–300 ml/day via the percutaneous tube placed operatively. The volume of the diet will depend on the capacity per back nutritional supplements used at each hospital. This volume will be increased to 400–600 ml/day on POD 2, and the volume from POD 3 will be decided by surgeons. Surgeons will select enteral formulas such as Elental® (EA pharma corporation), K-5S® (NUTRI corporation), and Pepucino® (Terumo corporation) based on their preference. The feeding jejunostomy catheter will be inserted through the jejunum wall and fixed using the Witzel Technique before closing the wound.

In both groups, oral solid feeding will start on POD 3. The total calories of food provided by hospitals are shown, and nurses will record the amount of food consumed by patients. Each treatment will be finished when patients’ oral food intake reaches to 60% of the nutritional requirement (25–30 kcal/day) [1]. The percutaneous tube will be removed at the bedside per the surgeon’s decision after the patient’s oral food intake attains the requirements.

### Criteria for discontinuing or modifying allocated interventions {11b}

Any patient requesting to end their participation in the study can be withdrawn from the study regardless of the stage they have reached in the study process. Patients do not have to provide the reason of withdrawal. Patients found to be pregnant or those judged ineligible to continue participating in the study by the investigators will also be withdrawn from the study.

In the EN group, the volume of the diet will be decreased if patients suffer from diarrhea that is not cured by antidiarrheal agents. TPN will be delivered if patients’ oral intake in the PPN group is less than 200 kcal/day on POD 14.

### Strategies to improve adherence to interventions {11c}

All treatments will be administered to participants during their stay in the hospital by attending surgeons; therefore, participants’ adherence to interventions is assured.

### Relevant concomitant care permitted or prohibited during the trial {11d}

All other treatments will be allowed.

### Provisions for post-trial care {30}

All patients who will suffer harm from trial participation will be covered by the Japanese public healthcare system and the insurance for this study.

### Outcomes {12}

The primary outcome of this study will be the infectious complication rate after PD. The secondary outcomes will be all postoperative complications; major complications (Clavien–Dindo grade > 3); clinically relevant POPF (CR-POPF); delayed gastric emptying (DGE); the length of hospital stay (LOS); time to functional recovery after surgery; serum levels of albumin, pre-albumin, and transferrin; the cost; side effects; and time to the introduction of adjuvant chemotherapy from surgery in patients with pancreatic cancer [14].

### Participant timeline {13}

**Fig. 2:**
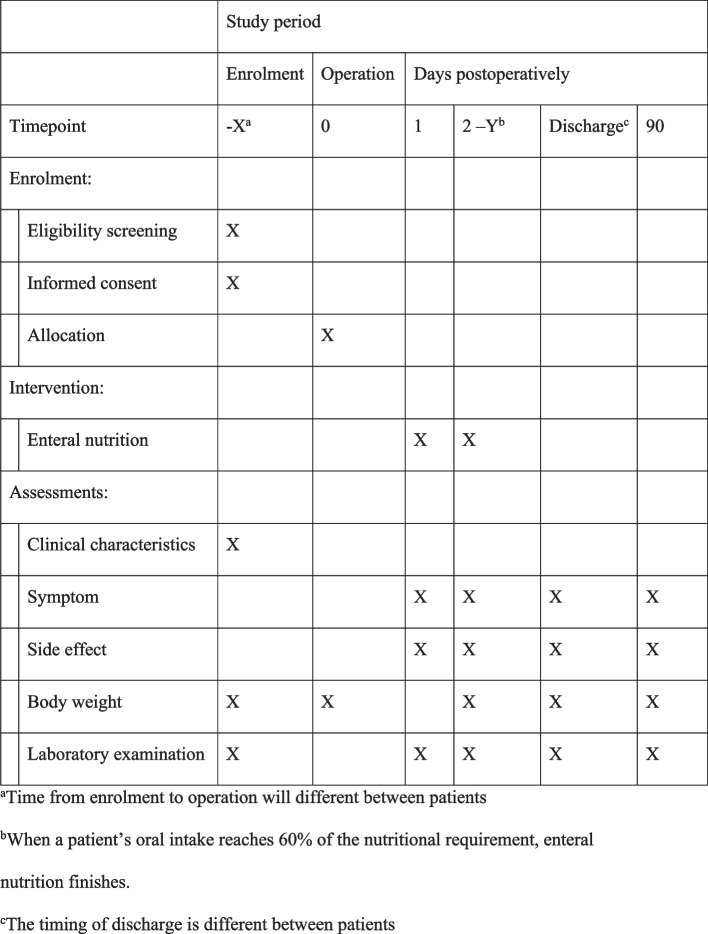
The schedule of enrolment, interventions, and assessments

The main timeline of this study is found in Fig. 2.

### Sample size {14}

The sample size was calculated for a 15% absolute reduction in the postoperative infectious complication rate in the EN group based on a postoperative complication rate of 40% in the PPN group [11]. To achieve 80% power and a 95% significance level, 152 patients are required in each group. To compensate for the loss of participants to follow-up (0.5%), we plan for 320 patients (160 in each group) to be included in the study.

### Recruitment {15}

This study will be conducted at six Japanese high-volume centers. The surgeons at each hospital will provide patients with adequate information about the study.

## Assignment of interventions: allocation

### Sequence generation {16a}

An electronic data capture system, Research Electronic Data Capture (REDCap), will be used to perform the randomization. Computerized stratified block randomization will be performed to determine the allocation to a treatment group in a 1:1 ratio. Stratification will be employed according to the hospital, age (≥ 70 or < 70), and preoperative diagnosis (pancreatic cancer or not) [3]. Data on all procedures will be managed by the Juntendo Clinical Research and Trial Center and will be unavailable to any researchers.

### Concealment mechanism {16b}

The result of the allocation will be shown via the REDCap system in each hospital.

### Implementation {16c}

The allocation sequence, enrolment of participants, and assignment of participants to interventions will be generated by the Juntendo Clinical Research and Trial Center.

## Assignment of interventions: blinding

### Who will be blinded {17a}

Data analysts will be blinded. Trial participants and researchers will not be blinded to the group allocations because the percutaneous tube for EN will be placed only in patients allocated to the EN group.

### Procedure for unblinding if needed {17b}

Not applicable.

## Data collection and management

### Plans for assessment and collection of outcomes {18a}

Case report forms are constructed as web-based forms (REDCap) and data entry will be conducted by surgeons or trained medical personnel. Baseline patient characteristics will include the age, sex, height, weight, hand grip strength, psoas mass index, comorbidity, preoperative diagnosis, preoperative treatment (neoadjuvant chemotherapy, antibiotics, biliary drainage, nutritional support), preoperative laboratory variables (white blood cell count, hemoglobin concentration, serum total protein, serum albumin, serum transferrin, serum pre-albumin, C-reactive protein), and the American Society of Anesthesiologists physical status.

Surgical data will include the surgical procedure, method of reconstruction, remnant pancreas texture, diameter of the main pancreatic duct, operation time, estimated blood loss, and need for a blood transfusion.

Postoperative data will include patients’ clinical conditions, morbidities and their severity, the mortality rate, length of hospital stay, time to functional recovery from surgery, laboratory data, cost, and any side effects. In patients with pancreatic cancer, the time to the introduction of adjuvant chemotherapy from surgery will also be measured. All patients will be followed up at each hospital for at least 90 days.

### Plans to promote participant retention and complete follow-up {18b}

Medical interviews and laboratory examinations will be booked for all patients at each hospital.

### Data management {19}

Every patient will be coded with an individual randomization number at each hospital and their data will be collected via an electric data system (REDCap). To enhance data quality, REDCap is designed so that researchers could not enter non-realistic values. Before data analyses, all data will be checked by an experienced research administrator independent of analyses.

### Confidentiality {27}

The form used to code patients will be stored in a locked cabinet with logged access only available to the researchers and administrators responsible for the study.

### Plans for collection, laboratory evaluation, and storage of biological specimens for genetic or molecular analysis in this trial/future use {33}

Not applicable.

## Statistical methods

### Statistical methods for primary and secondary outcomes {20a}

An intention-to-treat analysis will be conducted to compare primary and secondary outcomes between the EN group and the PN group. Differences between the groups of patients will be analyzed via Fisher’s exact test (as appropriate) for categorical data and by Student’s *t*-test for continuous variables. *P-*values of less than 0.05 will be considered statistically significant. Statistical analyses will be performed using SPSS version 28 (IBM Corp., Armonk, NY, USA).

### Interim analyses {21b}

Interim analyses are not planned.

### Methods for additional analyses (e.g., subgroup analyses) {20b}

Subgroup analyses are planned for baseline characteristics that might affect the primary endpoint. The pre-specified effect modification will include the disease (pancreatic cancer or not), age (≥ 70 or not), and preoperative biliary drainage.

### Methods in analysis to handle protocol non-adherence and any statistical methods to handle missing data {20c}

No statistical methods will be used to compensate for missing data.

### Plans to give access to the full protocol, participant-level data, and statistical code {31c}

Details of the full protocol, participant-level data, or statistical code will not be publicly available. Unpublished data will be made available upon reasonable request to the corresponding author of the publication.

## Oversight and monitoring

### Composition of the coordinating center and trial steering committee {5d}

Juntendo University will serve as the coordinating center. Only the investigators and members of the data center will have access to the anonymized data in REDCap.

### Composition of the data monitoring committee, its role and reporting structure {21a}

Juntendo Clinical Research and Trial Center will independently monitor the data. They have the responsibility of verifying patients’ eligibility, written informed consent, compliance with the protocol, and accuracy of the data in REDCap.

### Adverse event reporting and harms {22}

Researchers will immediately report serious adverse events (SAEs) associated with the trial to the chief investigator at each hospital. Then, the chief investigator will report SAEs to the director of the hospital and the principal investigator. The SAEs are shared with all researchers by the principal investigator. Data about all SAEs will also be collected in REDCap.

### Frequency and plans for auditing trial conduct {23}

An independent party will audit and report the results.

### Plans for communicating important protocol amendments to relevant parties (e.g., trial participants, ethical committees) {25}

Any protocol modifications will be reviewed by the Juntendo University Clinical Research Review Board and then registered at jRCT. All relevant information will be shared among the researchers.

### Dissemination plans {31a}

The results of this study will be published in a peer-reviewed journal and presented at national and international medical congresses.

## Discussion

In this RCT, we evaluate whether EN reduces the infectious complication rate after PD under the ERAS protocol. Patients start clear water without restrictions and solid food on POD 3, which is reasonable timing, as the ERAS protocol [15]. PPN combined with the early restart of oral intake is used as standard treatment. TPN will be used when patients in the PPN group could not get sufficient oral intake until POD 14 because TPN in the immediate postoperative period could increase the infectious complication rate and the overall morbidity according to the American Society for Parenteral and Enteral Nutrition (ASPEN) and European Society for Clinical Nutrition and Metabolism (ESPEN) guidelines [16, 17]. We have defined seven diseases as postoperative infectious complications (the primary endpoint) according to previous studies [3, 18, 19]. The diagnostic criteria for each complication are also clearly defined. On the other hand, inflammatory disorders of unknown causes will not be counted as infectious complications [20]. These definitions will provide clear evidence of the benefit of EN for patients undergoing PD. EN is delivered via percutaneous tubes placed operatively to increase its feasibility and accurately judge its efficacy. In a previous study conducted in France, EN delivered via nasojejunal tubes resulted in a high failure delivery rate of 37%, which made it difficult to assess the effect of EN [1]. Moreover, a previous systematic review reported that EN via percutaneous tubes was superior to that via nasojejunal tubes in the aspect of improving postoperative outcomes after PD [21]. The amount of feeding will be gradually increased to monitor the tolerance and to avoid patient drop-out. Patient allocation is determined by the hospital, age, and preoperative diagnosis. A patient’s age and preoperative diagnosis (pancreatic cancer or not) are reported to be associated with postoperative infectious complications [3]. We recognize that preoperative biliary drainage is a major risk factor for postoperative infectious complications; so, preoperative biliary drainage will serve as a factor in the planned subgroup analysis.

A potential limitation of this study is that we will not be able to perform a double-blind study, and this limitation could affect our results; however, it could be minimized by using strictly defined diagnostic criteria for infectious complications and the Clavien–Dindo grading system. Another limitation is that concomitant treatments could affect some outcomes. However, no treatments have strong evidence to mitigate the primary outcome of our study. Although our study will be performed under the ERAS protocol, the ERAS protocol is not completely the same in six hospitals because each hospital has its own ERAS protocol. However, we have an agreement in the majority of current ERAS items such as preoperative smoking and fasting, epidural analgesia, postoperative glycaemic control, early removal of drains, early and scheduled mobilization, and early diet from postoperative day 3 [15].

This trial is the first large-scale, well-designed RCT to investigate whether EN is beneficial for patients undergoing PD under the ERAS protocol. According to the results of this study, either EN or PPN would be adopted as standard nutritional support for patients undergoing PD.

## Trial status

Patient recruitment will begin on July 8, 2022. The recruitment will be completed by June 2024.

## Data Availability

The data that support the findings of this study will not be publicly available due to the need to protect the privacy of research participants; however, they will be available from the corresponding author on reasonable request.

## Abbreviations

ERAS: Enhanced recovery after surgery
EN: Enteral nutrition
PD: Pancreaticoduodenectomy
POD: Postoperative day
POPF: Postoperative pancreatic fistula
PPN: Peripheral parental nutrition
REDCap: Research Electronic Data Capture
RCT: Randomized controlled study
TPN: Total parental nutrition
